# Differential expression of Type I interferon and inflammatory genes in SARS‐CoV‐2‐infected patients treated with monoclonal antibodies

**DOI:** 10.1002/iid3.968

**Published:** 2023-10-13

**Authors:** Luca Maddaloni, Letizia Santinelli, Ginevra Bugani, Elio G. Cacciola, Alessandro Lazzaro, Chiara M. Lofaro, Sara Caiazzo, Federica Frasca, Matteo Fracella, Camilla Ajassa, Cristiana Leanza, Anna Napoli, Lilia Cinti, Aurelia Gaeta, Guido Antonelli, Giancarlo Ceccarelli, Claudio M. Mastroianni, Carolina Scagnolari, Gabriella d'Ettorre

**Affiliations:** ^1^ Department of Public Health and Infectious Diseases Sapienza University of Rome Rome Italy; ^2^ Virology Laboratory, Department of Molecular Medicine Sapienza University of Rome Rome Italy; ^3^ Laboratory of Microbiology and Virology, Department of Molecular Medicine Sapienza University of Rome Rome Italy; ^4^ Azienda Ospedaliero‐Universitaria Policlinico Umberto I Rome Italy

**Keywords:** interferon‐stimulated genes, monoclonal antibodies, SARS‐CoV‐2, Type I interferons

## Abstract

**Introduction:**

Considering the reported efficacy of monoclonal antibodies (mAbs) directed against the Spike (S) protein of severe acute respiratory syndrome coronavirus 2 (SARS‐CoV‐2) in reducing disease severity, the aim of this study was to investigate the innate immune response before and after mAbs treatment in 72 vaccinated and 31 unvaccinated SARS‐CoV‐2 patients.

**Methods:**

The mRNA levels of IFN‐I, IFN‐related genes and cytokines were evaluated using RT/real‐time quantitative PCR.

**Results:**

Vaccinated patients showed increased rate of negative SARS‐CoV‐2 PCR tests on nasopharyngeal swab compared with unvaccinated ones after mAbs treatment (*p* = .002). Unvaccinated patients had lower IFN‐α/ω and higher IFN‐related genes (IFNAR1, IFNAR2, IRF9, ISG15, ISG56 and IFI27) and cytokines (IL‐6, IL‐10 and TGF‐β) mRNA levels compared to vaccinated individuals before mAbs (*p* < .05 for all genes). Increased IFN‐α/ω, IFNAR1, IFNAR2 and IRF9 levels were observed in unvaccinated patients after mAbs treatment, while the mRNA expression ISGs and IL‐10 were reduced in all patients.

**Conclusion:**

These data suggest that anti‐S vaccinated patients have increased levels of innate immune genes compared to unvaccinated ones. Also, gene expression changes in IFN genes after mAbs administration are different according to the vaccination status of patients.

## Graphical abstract

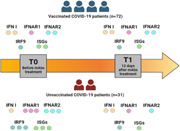

## 1 INTRODUCTION

Currently available vaccines and therapeutic approaches have proven useful in reducing coronavirus disease 2019 (COVID‐19)‐associated morbidity and mortality, and to moderate the impact of pandemic on healthcare resources.^1^ As a cornerstone resource, vaccine provides a stimulus for both humoral and cellular immune responses, required to clear infection and to maintain an immunological memory,^2^ but also monoclonal antibodies (mAbs) exhibit a great importance among the best available therapies. Distinct mAbs combinations, including casirivimab/imdevimab and bamlanivimab/etesevimab, received an Emergency Use Authorization from the US Food and Drug Administration for treatment of high‐risk outpatients recently diagnosed with mild‐to‐moderate COVID‐19, to reduce viral burden and prevent disease progression.^3^, ^4^ mAbs against severe acute respiratory syndrome coronavirus 2 (SARS‐CoV‐2) are designed to bind the receptor‐binding domain of Spike (S) protein, preventing the interaction with its receptor angiotensin converting enzyme 2 and entry into the host cell, and promoting its clearance by opsonization.^5^

Classically, virally infected cells produce Type I interferons (IFN‐I), which are involved in the early innate immune response.^6^ IFN‐I bind to their receptor (IFN‐α and ‐β receptor subunit 1 [IFNAR1] and 2 [IFNAR2]), in an autocrine and paracrine manner, and stimulate the phosphorylation and activation of the signal transducer and activator of transcription 1 (STAT1) and 2 (STAT2). When combined with the IFN regulatory factor 9 (IRF9), phosphorylated STAT1 and STAT2 form the IFN‐stimulated gene factor 3 complex, which migrates to the nucleous to promote the transcription of hundreds of interferon‐stimulated genes (ISGs). ISGs, in turn, inhibit virus multiplication at distinct levels, potentiate the innate immunity, and stimulate an adaptive response.^7^, ^8^ Several ISGs, such as ISG15, could be induced within the infected cell during acute virus infection even exploiting other ways independent from IFN signaling.^9^ As a consequence, distinct SARS‐CoV‐2 proteins are able to cause dysregulation on the IFN‐I production and IFN‐related genes, allowing virus to escape from such host defenses.^8^ Remarkably, one of the hallmarks of severe/critical form of COVID‐19 is the weak and delayed IFN‐I response along with an overproduction of both pro and anti‐inflammatory cytokines such as interleukin 1β (IL‐1β), 6 (IL‐6), and 10 (IL‐10), tumor necrosis factor α (TNF‐α), and transforming growth factor β (TGF‐β).^10^, ^11^, ^12^, ^13^ Numerous cytokines and chemokines induced by SARS‐CoV‐2 infection have been shown to be elevated after vaccination against SARS‐CoV‐2, although important differences with natural infection need to be considered. Indeed, upon vaccination the inflammatory cytokine response is early and transient, whereas during natural SARS‐CoV‐2 infection systemic cytokines levels remain elevated throughout COVID‐19 clinical course.^14^ Despite the well‐described efficacy and safety of mAbs therapy in SARS‐CoV‐2‐infected patients,^15^ the effect of this treatment on the IFN‐I pathway and inflammatory response is not yet known, nor whether vaccinated and unvaccinated individuals might display a different immunological response to mAbs.

Hence, the aim of this study was to evaluate whether differences exist in the virological response as well as in the levels of IFN‐I, IFN‐related genes, and cytokines genes between vaccinated and unvaccinated SARS‐CoV‐2‐infected patients after mAbs treatment. In particular, gene expression levels of IFN‐I (IFN‐α and IFN‐ω), IFN‐I receptor subunits (IFNAR1 and IFNAR2), IRF9, ISGs (ISG15, ISG56, IFN‐α‐inducible protein 27 [IFI27]), and cytokines (IL‐1β, IL‐6, IL‐10, TNF‐α, and TGF‐β) were examined in peripheral blood mononuclear cells (PBMCs) collected from SARS‐CoV‐2‐infected patients before and after mAbs treatment. Moreover, data on gene expression were evaluated according with the vaccination status and production of anti‐S antibodies.

## 2 MATERIALS AND METHODS

### 2.1 Participants

A longitudinal study at the Division of Infectious Diseases, Department of Public Health and Infectious Diseases, Umberto I Hospital of Sapienza University of Rome (Italy), including 116 patients with polymerase chain reaction (PCR) test confirmed SARS‐CoV‐2 infection on nasopharyngeal swab from April the 21 and December 10, 2021, discharged to home care, was conducted. All patients received exclusively therapeutic regimens based on combined mAbs, casirivimab (1200 mg) plus imdevimab (1200 mg), or bamlanivimab (700 mg) plus etesevimab (1400 mg), in a single administration, following italian drug agency statement. The data source for patient information analysis was derived from electronic medical records in the Hospital Electronic Information System. The following variables were considered for the study: age, gender, vaccination status against SARS‐CoV‐2, and Charlson Comorbidity Index.^16^ A healthy control group, composed of nine individuals matched by age and gender was also included. The study was approved by the Institutional Review Board (Department of Public Health and Infectious Diseases, Sapienza, University of Rome) and the Ethics Committee (Sapienza, University of Rome), and all study participants signed written informed consent.

### 2.2 PBMC isolation

Fresh peripheral blood samples (20 ml) were collected by venipuncture in Vacutainer tubes containing EDTA (BD Biosciences), from healthy individuals and SARS‐CoV‐2‐infected patients at baseline (at least 48 h from diagnosis) (T0) and almost 12 days after mAbs administration (T1), and were processed by Ficoll‐Hypaque density gradient centrifugation (Lympholyte, Cedarlane Labs) to obtain PBMCs. PBMCs were washed twice in phosphate‐buffered saline and stored at −80°C as dried pellets for RNA extraction.

### 2.3 Quantitative reverse transcriptase (RT)/real‐time PCR assays for messenger RNA (mRNA) expression levels

Quantitative RT/real‐time PCR for the analysis of *IFN‐α*, *IFN‐ω*, *IFNAR1*, *IFNAR2*, *IRF9*, *ISG15*, *ISG56*, *IFI27*, *IL‐1β*, *IL‐6*, *IL‐10*, *TNF‐α*, and *TGF‐β* mRNA levels were carried out with the LightCycler480 Instrument II (Roche), as previously described.^17^ Briefly, total RNA was extracted from PBMCs collected from SARS‐CoV‐2‐infected patients using a commercial RNA purification assay (ZymoBIOMICS RNA Miniprep Kit) and reverse‐transcribed using the High‐Capacity cDNA Reverse Transcription Kit (Applied Biosystems), according to the manufacturer's protocol. All primers and probes were added to the Probes Master Mix (Roche) at 500 and 250 nm, respectively, in a final volume of 70 μL. The *β‐glucuronidase* housekeeping gene was considered as an internal control. Gene expression values were calculated by the comparative Ct method. The primers and probe sequences used for *IFN‐α* (Hs. PT.58.24294810.g), *IFN‐ω* (Hs.PT.58.20160308.g), *IFN‐γ* (Hs.PT.58.3781960), *TNF‐α* (Hs.PT.58.45380900), *TGF‐β* (Hs.PT.58.39813975), *IL‐1β* (Hs.PT.58.1518186), *IL‐6* (Hs.PT.58.40226675), *IL‐10* (Hs.PT.58.2807216), *IFI27* (Hs.PT.58.1439222), *IFNAR1* (Hs.PT.58.20048943), *IFNAR2* (Hs.PT.58.1621113), and *IRF9* (Hs.PT.58.3264634) were purchased from Integrated DNA Technologies. The primers and probe sequences used for *ISG15* were the following: forward, 5′‐TGGCGGGCAACGAATT‐3′; reverse, 5′‐GGGTGATCTGCGCCTTCA‐3′; probe 5′‐(6FAM) TGAGCAGCTCCATGTC (TAM)‐3′. The primers and probe sequences used for *ISG56* were the following: forward, 5′‐TGAGAAGCTCTAGCCAACAACATGTC‐3′; reverse, 5′‐GAGCTTTATCCACAGAGCCTTTTC‐3′; probe 5′‐(6FAM) TATGTCTTTCGATATGCAGCCAAGTTTTACCG (TAM)‐3′.

### 2.4 Antibody titer against SARS‐CoV‐2 TrimericS protein quantification

Type G immunoglobulin (IgG) against SARS‐CoV‐2 Spike protein were determined in infected patients' serum using a commercial assay (LIAISON® SARS‐CoV‐2 TrimericS IgG). The assay provides anti‐S antibody titers as binding antibody units per ml (BAU/mL) and measures between 4.81 and 2080 BAU/mL. Values < 33.8 BAU/ml were considered negative according to the manufacturer's instructions. Specimens containing high levels of anti‐TrimericS IgG above the assay measuring range (>2080 BAU/mL) were automatically diluted with a factor of 1:10 using LIAISON® TrimericS IgG Diluent Accessory. In addition, anti‐S antibody titers were arbitrarily considered low between 33.8 and 400 BAU/mL, and high for values >400 BAU/mL.

### 2.5 Statistical analysis

Patients' data were expressed as median (interquartile range) or number (percentage). Demographic, virological, serological, and clinical patients' characteristics were analyzed using “N‐1” *χ* ^2^ test. Cross‐sectional data between vaccinated and unvaccinated patients were analyzed using the Mann–Whitney *U* test, whereas Wilcoxon signed‐rank test for paired samples was used to evaluate longitudinal data between T0 and T1. Spearman's *ρ* coefficient was calculated to assess the correlation between gene expression levels and vaccination induced antibody titers. A *p* < 0.05 was considered statistically significant. Statistical analyses were performed using GraphPad Prism software, version 9.4 (GraphPad Software Inc.).

## 3 RESULTS

### 3.1 Study population

A total of 116 SARS‐CoV‐2‐infected patients were enrolled in the study. Among them, 103 completed the follow‐up (collection of paired blood samples at T0 and T1). Demographic and clinical characteristics of SARS‐CoV‐2‐infected patients before mAbs treatment (T0) are reported in Table 1; SARS‐CoV‐2‐vaccinated patients were older, displayed higher Charlson Comorbidity Index and anti‐S antibody levels before mAbs treatment and lower SARS‐CoV‐2‐RNA levels after treatment compared to unvaccinated ones. Type of anti‐SARS‐CoV‐2 vaccine and number of vaccine doses previously received at the time of mAbs administration are described in Supporting Information: Table 1. In particular, 61.2% (63/103) of patients received casirivimab plus imdevimab therapy, whereas 38.8% (40/103) of them were treated with bamlanivimab plus etesevimab. Data on SARS‐CoV‐2‐RNA 12 days after mAbs treatment (T1) were available for 85 patients. SARS‐CoV‐2‐RNA levels measured in nasopharyngeal swab were defined as undetectable (*C*_t_ value > 45) or high and low for *C*_t_ values < 34 and ≥34, respectively, as previously described.^18^ The vaccinated patient's group had increased negative rate of RT‐PCR SARS‐CoV‐2 tests compared with unvaccinated ones after mAbs treatment (T1, *p* = .002). After mAbs treatment, the number of patients with high SARS‐CoV‐2‐RNA levels (*C*_t_ values ≥ 34) was higher in the unvaccinated individuals compared with the vaccinated ones ([*n* = 15; 58%] vs. [*n* = 19; 32%]; *p* = .025) (Table 1). Data on antibody titers against S protein, measured at T0, were available only for 95 patients: 23 out of 95 (24.2%) patients exhibited high antibody titer (>400 BAU/mL), 33 out of 95 (34.7%) low anti‐S antibody titer (≥33.8 to ≤400 BAU/mL), whereas 39 out of 95 (41.1%) had a negative anti‐S antibody test result (<33.8 BAU/mL). Moreover, 13 out of 64 (20.3%) vaccinated patients had negative antibody titer (<33.8 BAU/mL), whereas 5 out of 31 (16.1%) unvaccinated patients had detectable levels of antibodies (≥33.8 BAU/mL) (Table 1). The percentage of patients with undetectable anti‐S antibodies (*n* = 18; 58%) with SARS‐CoV‐2‐RNA levels <34 *C*_t_ was higher compared with those with low (*n* = 8; 30.8%) or high (*n* = 5; 27%) titers of anti‐S antibodies (*p* = .0418 and *p* = .0158, respectively). Furthermore, those patients with high anti‐S antibody titer had increased negative rate of RT‐PCR SARS‐CoV‐2 tests compared with negative anti‐S antibody titers ones ([*n* = 8; 38.1%] vs. [*n* = 4; 13%]; *p* = .037]) (Supporting Information: Table 2).

**Table 1:** Demographic, virological, serological, and clinical characteristics of SARS‐CoV‐2‐infected patients.

	SARS‐CoV‐2‐vaccinated patients (72/103, 70%)		SARS‐CoV‐2‐unvaccinated patients (31/103, 30%)		
Parameters	Median (IQR 25%–75%)	*n* (%)	Median (IQR 25%–75%)	*n* (%)	*p*
Sex assigned at birth					
Male	–	36 (50)	–	14 (45)	.643
Female		36 (50)		17 (55)	.435
Age (years)	66 (58–75)	–	57 (46–66)	–	**.004**
Charlson Comorbidity Index*	3 (3–4)	–	2 (1–3)	–	**.006**
Anti‐Spike (S) antibody levels at T0^a^					
Undetectable (<33.8 BAU/mL)	–	13 (20.3)	–	26 (83.9)	**<.0001**
Low (33.8–400 BAU/mL)	–	30 (46.9)	–	3 (9.7)	**.0004**
High (>400 BAU/mL)	–	21 (32.8)	–	2 (6.4)	**.0051**
SARS‐CoV‐2‐RNA levels at T1^b^					
High (<34 *C*_t_)	–	19 (32)	–	15 (58)	**.025**
Low (≥34 *C*_t_)	–	23 (39)	–	11 (42)	.796
Undetected (>45 *C*_t_)	–	17 (29)	–	0 (0)	**.002**

*Note*: Data were analyzed using “N‐1” *χ* ^2^ test and *p* < .05 were considered statistically significant.

Abbreviations: BAU, binding antibody units; IQR, interquartile range; mAbs, monoclonal antibodies; SARS‐CoV‐2, severe acute respiratory syndrome coronavirus 2.

^a^ Before mAbs therapy. Data available for 64 and 31 vaccinated and unvaccinated patients, respectively.

^b^ 12 days after mAbs therapy. Data available for 59 and 26 vaccinated and unvaccinated patients, respectively.

*The Charlson Comorbidity Index predicts the mortality for a patient who may have a range of concurrent conditions.^16^

### 3.2 Levels of IFN‐I, IFN‐related genes, and cytokines in SARS‐CoV‐2‐infected patients before mAbs treatment according to the vaccination status

**Figure 1:**
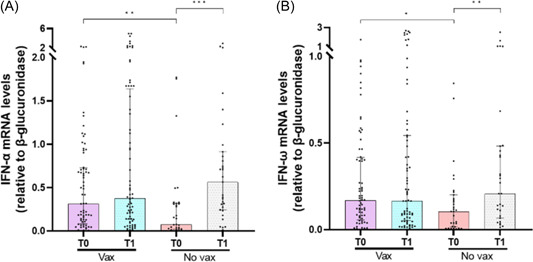
Comparison of interferon‐α (IFN‐α) (A) and IFN‐ω (B) messenger RNA (mRNA) expression levels before (T0) and 12 days after monoclonal antibodies (mAbs) treatment (T1) between vaccinated (vax) and unvaccinated (No vax) severe acute respiratory syndrome coronavirus 2‐infected patients. Data were analyzed using the Mann–Whitney *U* test for unpaired samples and the Wilcoxon signed‐rank test for paired samples. *p < .05; **p < .01; ***p < .001.

**Figure 2:**
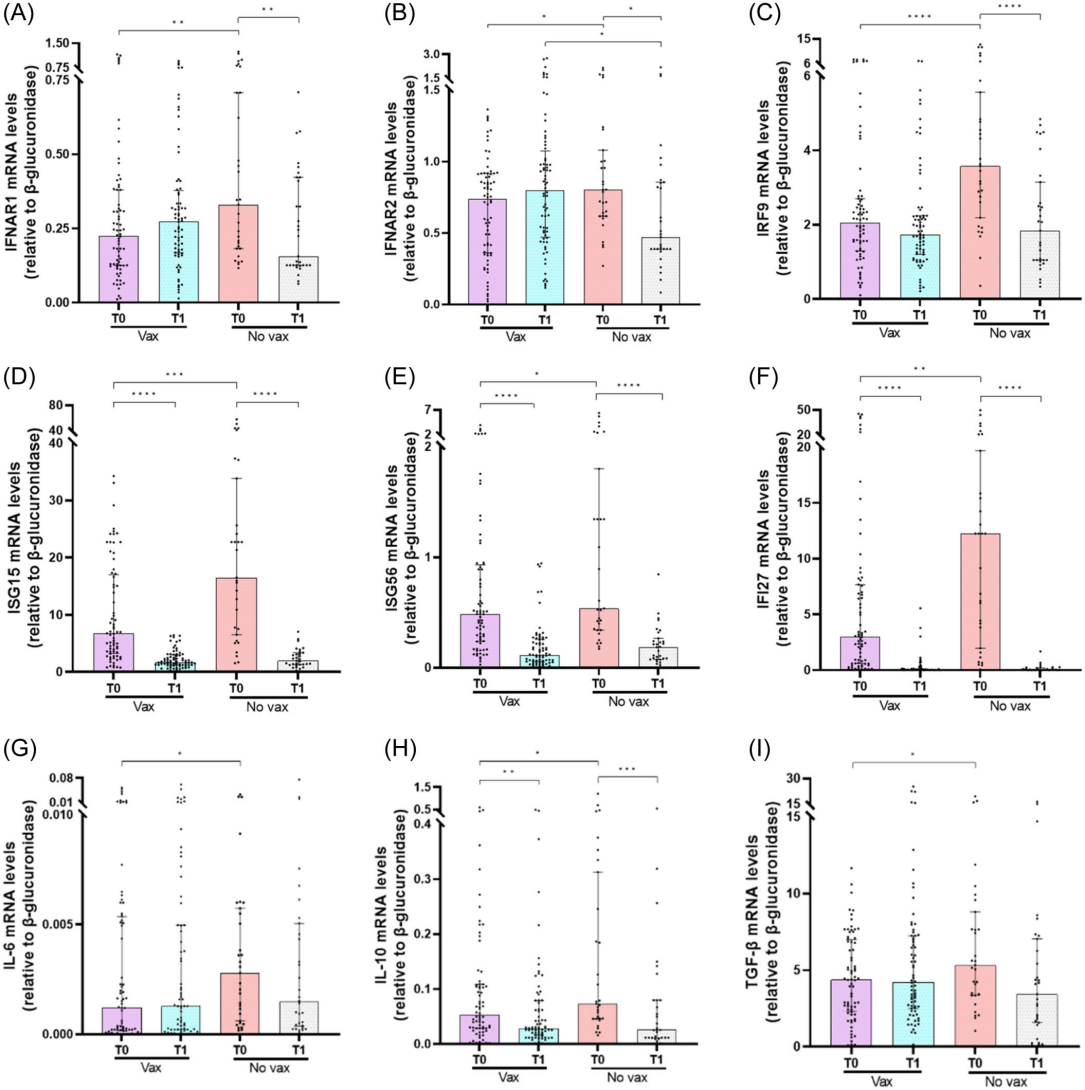
Comparison of interferon (IFN)‐α and ‐β receptor subunit 1 (IFNAR1) (A), IFNAR2 (B), IFN regulatory factor 9 (IRF9) (C), interferon‐stimulated gene 15 (ISG15) (D), interferon‐stimulated gene 56 (ISG56) (E), IFN‐α‐inducible protein 27 (IFI27) (F), interleukin (IL)‐6 (G), IL‐10 (H), transforming growth factor‐β (TGF‐β) (I) messenger RNA (mRNA) expression levels before (T0) and 12 days after monoclonal antibodies (mAbs) treatment (T1) between vaccinated (vax) and unvaccinated (no vax) severe acute respiratory syndrome coronavirus 2 (SARS‐CoV‐2)‐infected patients. Data were analyzed using the Mann–Whitney *U* test for unpaired samples and the Wilcoxon signed‐rank test for paired samples. *p < .05; **p < .01; ***p < .001; ****p < .0001.

To investigate whether previous SARS‐CoV‐2 vaccination resulted in changes in the expression levels of IFN‐I, IFN‐related genes, and cytokines genes, SARS‐CoV‐2‐infected patients were stratified according to their vaccination status. Unvaccinated patients had lower mRNA levels of *IFNα* (*p* = .0064) and *IFN‐ω* (*p* = .0342) compared with vaccinated ones (Figure 1A,B). By contrast, unvaccinated patients exhibited higher mRNA levels of IFN‐I receptor subunits (*IFNAR1* [*p* = .0094], *IFNAR2* [*p* = .033], *IRF9* [*p* < .0001]), ISGs (*ISG15* [*p* = .0008], *ISG56* [*p* = .0361], *IFI27* [*p* = .0041]), and distinct cytokines (*IL‐6* [*p* = .047], *IL‐10* [*p* = .0337], *TGF‐β* [*p* = .0442]) (Figure 2A–I). Also, *IL‐1β* and *TNF‐α* transcript levels were similar between the two groups of SARS‐CoV‐2‐positive patients (Supporting Information: Figure 1A,B).

### 3.3 Levels of IFN‐I, IFN‐related genes, and cytokines in SARS‐CoV‐2‐infected patients before mAbs treatment according with the anti‐S antibody titer

**Figure 3:**
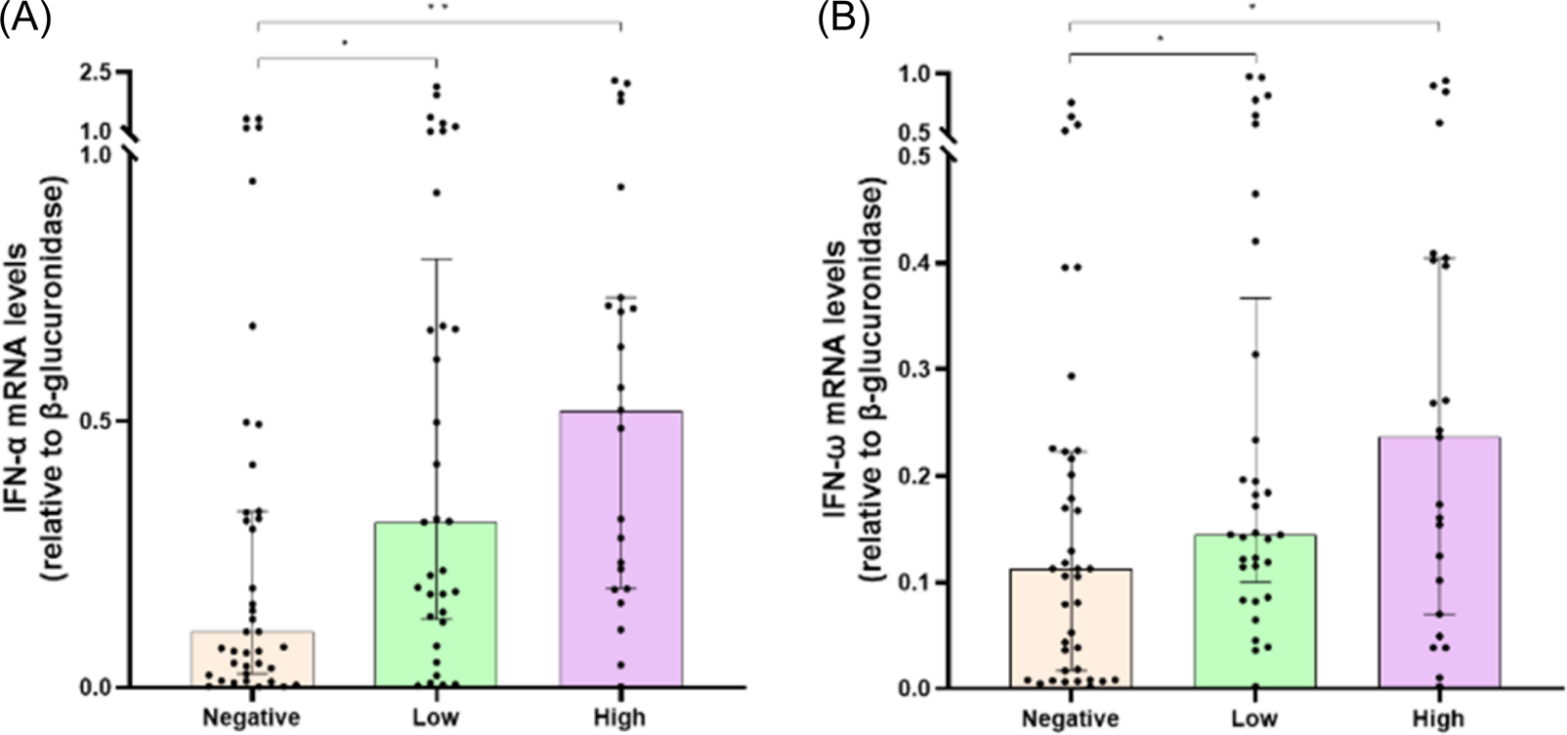
Comparison of interferon‐α (IFN‐α) (A) and IFN‐ω (B) messenger RNA (mRNA) expression levels before (T0) monoclonal antibodies (mAbs) treatment between severe acute respiratory syndrome coronavirus 2‐infected patients with high (>400 BAU/mL), low (≥33.8 to ≤400 BAU/mL), or negative (<33.8 BAU/mL) anti‐S antibody titers. Data were analyzed using the Mann–Whitney *U* test. *p < .05; **p < .01.

**Figure 4:**
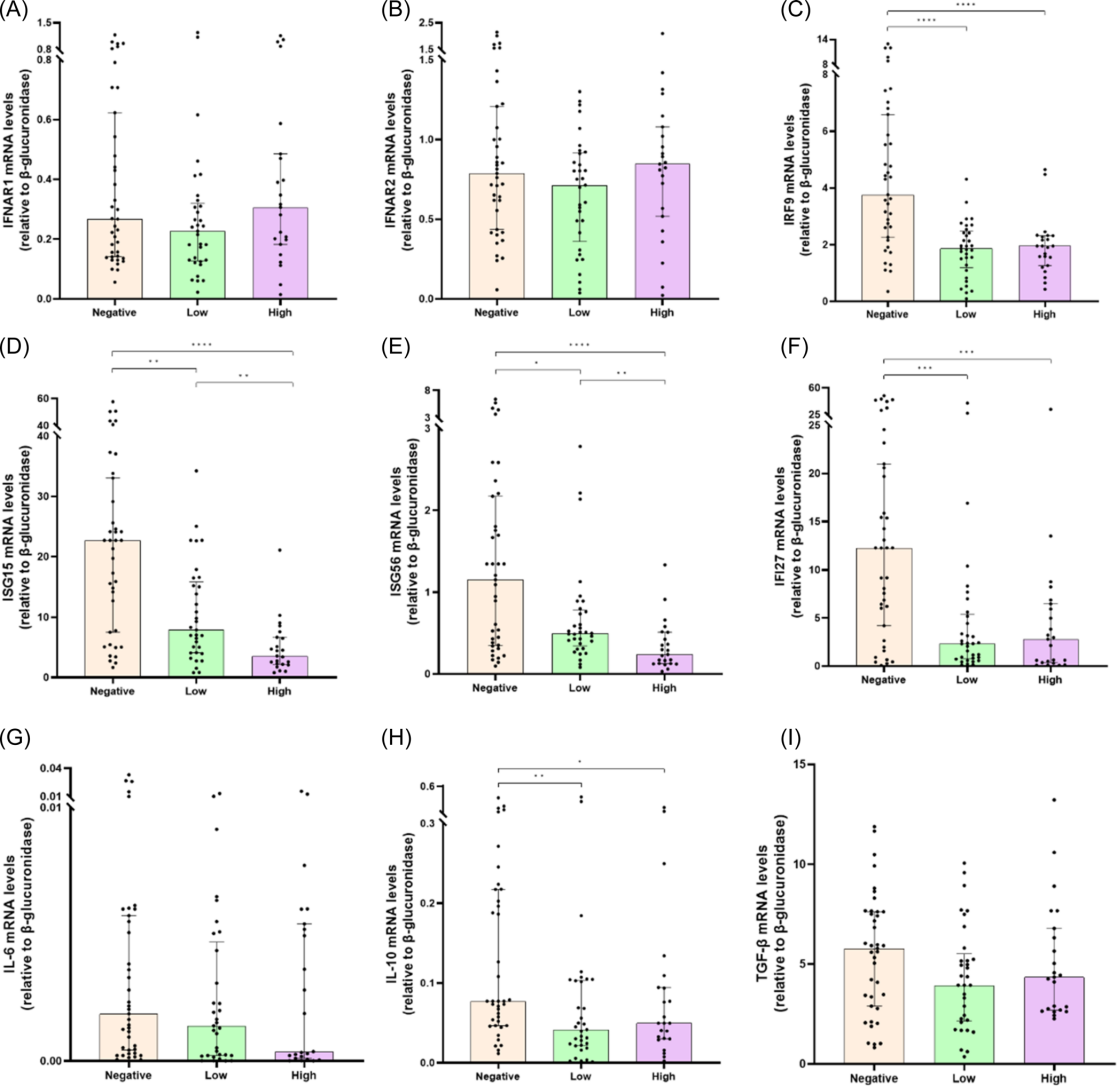
Comparison of interferon (IFN)‐α and ‐β receptor subunit 1 (IFNAR1) (B), IFNAR2 (B), IFN regulatory factor 9 (IRF9) (C), IFN‐stimulated gene 15 (ISG15) (D), IFN‐stimulated gene 56 (ISG56) (E), IFN‐α‐inducible protein 27 (IFI27) (F), interleukin‐6 (IL‐6) (G), interleukin‐10 (IL‐10) (H), transforming growth factor‐β (TGF‐β) (I) messenger RNA (mRNA) expression levels before (T0) monoclonal antibodies (mAbs) treatment between severe acute respiratory syndrome coronavirus 2 (SARS‐CoV‐2)‐infected patients high (>400 BAU/mL), low (≥33.8 to ≤400 BAU/mL), or negative (<33.8 BAU/mL) anti‐S antibody titers. Data were analyzed using the Mann–Whitney *U* test. *p < .05; **p < .01; ***p < .001; ****p < .0001.

The relationship between titer of anti‐S antibodies and innate immune response in SARS‐CoV‐2‐infected patients was evaluated. *IFN‐α* (*r* = .2586; *p* = .0183) transcript levels were positively correlated with the anti‐S antibody titers, whereas *IRF9* (*r* = −0.2293; *p* = .37), *ISG15* (*r* = −0.5327; *p* = .0001), *ISG56* (*r* = −0.4635; *p* < .0001), *IFI27* (*r* = −0.394; *p* = .0002), and *IL‐10* (*r* = −0.2201; *p* = .0456) mRNAs were negatively correlated (Table 2). No significant correlations were found between anti‐S antibodies levels and *IFN‐ω*, *IFNAR1*, *IFNAR2*, *IL‐6*, and *TGF‐β* mRNAs (Table 2). Gene expression analysis showed that patients with low and high anti‐S antibody titers had higher *IFN‐α* (*p* = .0294 and *p* = .0014, respectively) and *IFN‐ω* (*p* = .0275 and *p* = .0267, respectively) mRNA levels than those without anti‐S antibodies (Figure 3A,B). On the other hand, anti‐S antibodies negative patients had increased mRNA amount of distinct immune markers compared to those with low or high anti‐S antibodies levels: *IRF9* (*p* < .0001 for both groups), *ISG15* (*p* = .001 and *p* < .0001, respectively), *ISG56* (*p* = .0191 and *p* < .0001, respectively), *IFI27* (*p* = .002 for both groups), *IL‐10* (*p* = .0028 and *p* = .0473, respectively) (Figure 4C–F,H). Comparable levels of other tested genes (*IFNAR1*, *IFNAR2*, *IL‐6*, and *TGF‐β*) were observed between the three groups (Figure 4A,B,G,I).

**Table 2:** Correlation between anti‐Spike (S) antibody titers and IFN‐α, IFN‐ω, IFNAR1, IFNAR2, IRF9, ISG15, ISG56, IFI27, IL‐6, IL‐10, and TGF‐β mRNA levels before mAbs treatment (T0).

		IFN‐α	IFN‐ω	IFNAR1	IFNAR2	IRF9	ISG15	ISG56	IFI27	IL‐6	IL‐10	TGF‐β
Anti‐S antibody titers	*r*	.2586	.1564	.0426	.0404	−.2293	−.5327	−.4635	−.3940	−.0707	−.2201	−.0807
	*p*	**.0183**	.1580	.7024	.7166	**.0370**	**<.0001**	**<.0001**	**.0002**	.5252	**.0456**	.4681

*Note*: Data were analyzed using Spearman *r* correlation test and *p* < .05 were considered statistically significant.

Abbreviations: IFI27, IFN‐α‐inducible protein 27; IFN‐α, interferon‐α; IFN‐ω, interferon‐ω; IFNAR1, IFN‐α and ‐β receptor subunit 1; IFNAR2, IFN‐α and ‐β receptor subunit 1; IL‐6, interleukin‐6; IL‐10, interleukin‐10; IRF9, interferon regulatory factor 9; ISG15, interferon‐stimulated gene 15; ISG56, interferon‐stimulated gene 56; TGF‐β, transforming growth factor‐β; mAbs, monoclonal antibodies; mRNA, messenger RNA.

### 3.4 Levels of IFN‐I, IFN‐related genes, and cytokines in unvaccinated and vaccinated SARS‐CoV‐2‐infected patients after mAbs administration

Changes in IFN‐I, IFN‐related genes, and cytokines levels were evaluated in PBMCs of SARS‐CoV‐2‐infected patients after mAbs treatment according to the vaccination status. Unvaccinated patients had higher transcript levels for *IFN‐α* (*p* = .0006) and *IFN‐ω* (*p* = .0027) (Figure 1A,B) 12 days after mAbs treatment (T1) compared with baseline (T0), whereas *IFNAR1* (*p* = .0091), *IFNAR2* (*p* = .0167), and *IRF9* (*p* < .0001) (Figure 2A,C) mRNA levels were reduced. By contrast, no significant changes were found for *IFN‐α*, *IFN‐ω*, *IFNAR1*, *IFNAR2*, and *IRF9* mRNAs in vaccinated patients after mAbs treatment (Figures 1A,B and 2A–C). mAbs treatment also promoted a reduction in transcript levels of *ISG15* (*p* < .0001 for both groups), *ISG56* (*p* < .0001 for both groups), and *IFI27* (*p* < .0001 for both groups) in both vaccinated and unvaccinated patients (Figure 2D–F). Moreover, *IL‐10* mRNA was reduced in both groups after mAbs treatment (*p* = .009 for vaccinated; *p* = .0003 for unvaccinated), whereas *IL‐6*, *TGF‐β*, *IL‐1β*, and *TNF‐α* transcript levels were similar between T0 and T1 (Figure 2G–I and Supporting Information: Figure 1A,B).

## 4 DISCUSSION

To date vaccines remain the best weapon to fight a pandemic viral infection, as we observed recently with SARS‐CoV‐2.^19^ Therapy with mAbs has been proposed for high‐risk SARS‐CoV‐2‐infected individuals to prevent progression to severe COVID‐19 and reduce hospitalization.^5^ In this study, we evaluated the expression levels of IFN‐I, IFN‐related genes and various cytokines in patients before and after mAbs treatment according to the anti‐S vaccination status. First, a considerable number (29%) of SARS‐CoV‐2‐vaccinated patients tested negative to SARS‐CoV‐2 RT‐PCR 12 days after mAbs therapy, whereas all unvaccinated patients remained positive and most of them (58%) had *C*_t_ values of SARS‐CoV‐2‐RNA < 34. In agreement, most of patients with high SARS‐CoV‐2‐RNA levels (*C*_t_ values ≥ 34) had undetectable anti‐S antibodies, whereas an increased rate of negative SARS‐CoV‐2‐RNA tests was observed in those patients with high amount of anti‐S antibodies. As a first step for gene expression analysis, we performed a comparison between SARS‐CoV‐2 patients and healthy donors. This analysis showed that mRNA levels of some genes were slightly higher in patients than in healthy donors, while other genes had similar levels between the two groups (Supporting Information: Figure 2). These results can be in part explained considering that our patients had a clinical symptomatology from absent to mild, so the production of IFN pathways and cytokines might be not highly dysregulated as reported in patients with severe COVID‐19.

Interestingly, we found that IFN‐α and IFN‐ω mRNA levels were reduced in unvaccinated compared to vaccinated individuals before mAbs treatment. These differences might be due to a potential IFN‐I protection role observed in vaccinated subjects, as suggested for yellow fever vaccine.^20^ Indeed, a previous anti‐S vaccination might permit a better control of early virus replication allowing IFN‐I to act immediately against SARS‐CoV‐2 and avoiding the viral escape mechanisms developed to overcome innate immunity. By contrast, IFNAR1 and IFNAR2 mRNA levels were increased in unvaccinated individuals compared with vaccinated ones. In our opinion, in vaccinated patients the IFN response appears to be ready, controlled, and driven by the effects of the vaccine, as suggested previously.^21^ In unvaccinated patients, the expression of different components of IFN pathway seems to be altered; in particular, the host inability to efficiently contrast the early stages of SARS‐CoV‐2 infection could allow the virus to inhibit IFN‐I production^8^ and promote the IFN receptors disruption. In agreement, Hayn et al.^22^ found that viral protein nsp14 reduce in vitro IFNAR1 expression on surface of SARS‐CoV‐2‐infected cells, favoring its lysosomal degradation. Therefore, the increased gene expression of IFNAR1/2 observed in unvaccinated patients might be an immune defense mechanism of the infected cell to physiologically subvert the reduction in the expression of receptor caused by SARS‐CoV‐2. Furthermore, an increase in IFNAR1/2 expression might allow that, although IFN‐1 levels are reduced, more receptor ligand interactions could form, increasing the production of several ISGs. Notably, the unvaccinated patient's group exhibited higher mRNA levels of IRF9, ISG15, ISG56, and IFI27, despite reduced IFN‐I production compared with vaccinated ones. In this context, IFN‐independent stimulation of these ISGs, might be due to the SARS‐CoV‐2 itself or mediated by the action of other cytokines.^9^ In this complex scenario, unvaccinated patients had increased IL‐6, IL‐10, and TGF‐β mRNA levels before mAbs treatment, suggesting a state of major inflammation during SARS‐CoV‐2 infection compared with the vaccinated ones. In support of this hypothesis, IL‐6 is one of the key mediators of inflammation and is considered a central mediator of toxicity in cytokine release syndrome,^23^ a major cause of fatal outcome in COVID‐19.^24^ Despite the well‐known anti‐inflammatory properties of IL‐10, evidence from literature showed a COVID‐19 severity prediction role for this cytokine, together with the pro‐inflammatory IL‐6.^25^, ^26^ In addition, TGF‐β is an anti‐inflammatory cytokine produced by most immune cells during SARS‐CoV‐2 infection, but an adequate immune response is inhibited by its overproduction, as reported in several respiratory viral infections.^27^, ^28^

To further assess whether the vaccination status might modulate the innate immune response, SARS‐CoV‐2‐infected patients were stratified according to anti‐S antibody production. We found that anti‐S antibody‐positive patients exhibited increased IFN‐α and IFN‐ω mRNA levels than anti‐S antibody‐negative ones. A positive correlation was found between anti‐S antibody titers and IFN‐α mRNA production, indicating that anti‐S vaccine induce adaptive immunity, but also innate response as previously reported.^29^ At the same time, anti‐S antibody‐positive patients had lower IRF9, ISG15, ISG56, IFI27, and IL‐10 mRNA levels than anti‐S antibody‐negative ones, with asserted negative correlation between these genes and antibody titers. Given these findings, as anti‐S antibody levels decreased, the expression of almost immune and inflammatory genes analyzed in this study appears to be closer to that of unvaccinated patients.

As far as the analysis of the effects of mAbs on immune response was concerned, the transcript levels of IFN‐α and IFN‐ω did not change after mAbs treatment in vaccinated patients, whereas unvaccinated group showed increased levels of these genes, which reached levels similar to those of the vaccinated one. Probably the SARS‐CoV‐2‐RNA clearance caused by mAbs therapy in unvaccinated patients could have promoted IFN‐I response at levels similar to those of vaccinated patients. In this regard, IFNAR1, IFNAR2, and IRF9 mRNAs were reduced in the unvaccinated group at T1, whereas the mAbs treatment did not result in any relevant changes in the expression of these genes in the vaccinated one. Moreover, mAbs treatment decreased ISG15, ISG56, IFI27, and IL‐10 mRNA in both groups. Therefore, these results confirm that mAbs treatment favor the SARS‐CoV‐2‐RNA disappearance and suggest that this therapy might participate indirectly in the modulation of the expression of IFN‐I, IFN‐related gene, and the inflammation status in unvaccinated patients, whereas the expression of most of the immune genes analyzed, with the exception of ISGs and IL‐10 mRNAs, could be attenuated and independent from mAbs treatment in vaccinated patients. Given the enhanced innate immune response observed after vaccination,^30^ and these results, we speculated that anti‐S vaccinated patients are immunological advantaged compared with unvaccinated patients, who are able to reach the immune status of the former group in term of expression of IFN and cytokines genes only after mAbs administration. Data from literature showed a decrease in IFN‐I protein levels in unvaccinated patients 5 days after casirivimab/imdevimab treatment.^31^ However, compared with our study design, there were differences in mAbs administration timing and follow‐up analysis. Moreover, levels of IFN‐I were measured as proteins and nor as mRNAs.

From a clinical point of view, a previous study found that mAbs therapy with casirivimab/imdevimab was associated with a low rate of hospitalization in both vaccinated and unvaccinated individuals.^32^ Although IFN‐I mRNA expression did not change in our vaccinated patients after mAbs treatment, a relevant number of patients had undetectable SARS‐CoV‐2 RNA, maybe due to the combined antiviral effect of previous vaccination and mAbs. By contrast, despite the increased IFN‐I mRNA expression levels in the unvaccinated group following mAbs treatment, none were negative for SARS‐CoV‐2‐RNA 12 days after the treatment. A recent study reported that high‐risk SARS‐CoV‐2 patients treated with nirmatrelvir/ritonavir had more rapid conversion from positive to negative SARS‐CoV‐2 RT‐PCR test compared with nontreated patients, without significant differences based on the vaccination status.^33^

## 5 CONCLUSIONS

Our findings confirm that mAbs treatment is effective against SARS‐CoV‐2 infection, reducing viral RNA to low or undetectable levels 12 days after mAbs treatment, mostly in those patients that have received a previous anti‐S vaccination. Furthermore, mAbs treatment promotes an increase in the IFN‐α/ω levels and IFN‐related genes reduction in SARS‐CoV‐2‐infected unvaccinated patients, restoring them to the expression levels found in vaccinated patients before treatment.

## 6 LIMITATION OF THE STUDY

Despite the significant outcomes in the evaluation of innate immunity in mAbs treated SARS‐CoV‐2 patients, this study has some limitations. SARS‐CoV‐2 vaccines showed to differ each other in term of safety and efficacy, evaluated in adverse events and neutralizing antibodies production, respectively,^34^ but most of our patients were vaccinated with BNT162b2 and the other vaccines percentages were too small to assess eventual differences. We only evaluated IFN, IFN‐related genes, and cytokines mRNA expression, whereas it would have been interesting to evaluate them at protein level. The evaluation of mAbs effect on IgG subclasses would also have been very interesting, but, unfortunately, the serological test used does not allow discrimination of IgG subclasses. Moreover, further analysis based on cellular immunity are needed to better define the immunological effects of mAbs treatment in SARS‐CoV‐2‐infected patients.

## AUTHOR CONTRIBUTIONS

**Luca Maddaloni**: Data curation (lead); formal analysis (lead); methodology (equal); project administration (equal); writing—original draft (lead); writing—review & editing (equal). **Letizia Santinelli**: Methodology (equal); writing—review & editing (equal). **Ginevra Bugani**: Methodology (equal). **Elio G. Cacciola**: Resources (equal). **Alessandro Lazzaro**: Resources (equal). **Chiara M. Lofaro**: Methodology (equal). **Sara Caiazzo**: Methodology (equal). **Federica Frasca**: Methodology (equal); writing—review & editing (equal). **Matteo Fracella**: Methodology (equal); writing—review & editing (equal). **Camilla Ajassa**: Resources (equal). **Cristiana Leanza**: Resources (equal). **Anna Napoli**: Methodology (equal). **Lilia Cinti**: Methodology (equal). **Aurelia Gaeta**: Validation (equal); visualization (equal). **Guido Antonelli**: Funding acquisition (equal); validation (equal); visualization (equal). **Giancarlo Ceccarelli**: Validation (equal); visualization (equal); writing—review & editing (equal). **Claudio M. Mastroianni**: Funding acquisition (equal); validation (equal); visualization (equal). **Carolina Scagnolari**: Conceptualization (equal); funding acquisition (equal); investigation (equal); project administration (equal); supervision (equal); validation (equal); visualization (equal); writing—review & editing (equal). **Gabriella d'Ettorre**: Conceptualization (equal); funding acquisition (equal); investigation (equal); project administration (equal); supervision (equal); validation (equal); visualization (equal); writing—review & editing (equal).

## CONFLICT OF INTEREST STATEMENT

The authors declare no conflict of interest.

## ETHICS STATEMENT

The study was approved by the institutional review board (Department of Public Health and Infectious Diseases, Sapienza, University of Rome) and the Ethics Committee (Sapienza, University of Rome), and all study participants signed written informed consent.

## Supporting information

Supporting Information.

## Data Availability

Correspondence and requests for materials should be addressed to Giancarlo Ceccarelli (https://orcid.org/0000-0001-5921-3180).
