# Supplementary material for: Differential expression of Type I interferon and inflammatory genes in SARS‐CoV‐2‐infected patients treated with monoclonal antibodies
Source: Immun Inflamm Dis. 2023 Oct 13;11(10):e968. doi: 10.1002/iid3.968 (PMC10571496; doi:10.1002/iid3.968)
Supplement: Supplementary file 1 — Supporting Information. [file IID3-11-e968-s001.docx]

**Supplementary Table 1** SARS-CoV-2 vaccination status of study population before mAbs treatment (T0).

| SARS-COV-2 vaccinated patients | |
| --- | --- |
| **Vaccine type** | **n (%)** |
| • Comirnaty (BNT162b2) | 63/72 (87.5%) |
| 2 doses | 49/63 (77.8%) |
| 1 dose | 14/63 (22.2%) |
| • Spikevax (mRNA-1273) | 4/72 (5.55%) |
| 2 doses | 4/4 (100%) |
| 1 dose | 0/4 (0%) |
| • Vaxzevria (ChAdOx1-S) | 4/72 (5.55%) |
| 2 doses | 3/4 (75%) |
| 1 dose | 1/4 (25%) |
| • Janssen (Ad26.COV2.S) | 1/72 (1.4%) |

**Supplementary Table 2** Differences in SARS-CoV-2-RNA levels after mAbs therapy between patients with undetectable (< 33.8 BAU/ml), low (33.8 BAU/ml - 400 BAU/ml) and high (> 400 BAU/ml) anti-Spike (S) antibody titers.

|  | | Undetectable anti-S Ab titer patients (31/78, 39.7%) (A) | Low anti-S Ab titer patients (26/78, 33.3%) (B) | High anti-S Ab titer patients (21/78, 27%) (C) |  |  |  |
| --- | --- | --- | --- | --- | --- | --- | --- |
| **SARS-CoV-2-RNA levels at T1** | **n (%)** | | **n (%)** | **n (%)** | **A vs B p values** | **A vs C p values** | **B vs C p values** |
| High (<34 Ct) | 18 (58) | | 8 (30.8) | 5 (23.8) | **0.0418** | **0.0158** | 0.5978 |
| Low (≥34 Ct) | 9 (29) | | 14 (53.8) | 8 (38.1) | 0.0595 | 0.4966 | 0.2887 |
| Undetected (>45 Ct) | 4 (13) | | 4 (15.4) | 8 (38.1) | 0.7971 | **0.037** | 0.0792 |

Data were analyzed using "N-1" Chi-squared test and p < 0.05 were considered statistically significant.


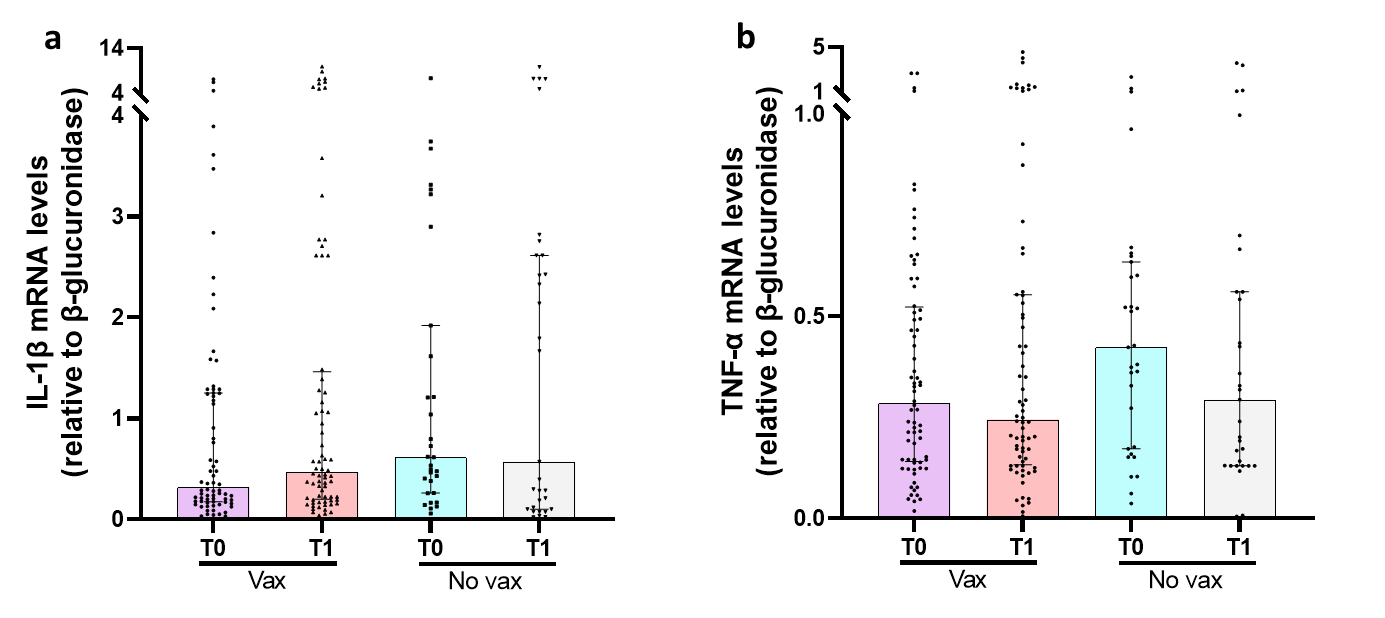


**Supplementary Figure 1** Comparison of IL-1β **(a)** and TNF-α **(b)** mRNA expression levels before (T0) and 12 days after mABs treatment (T1) between vaccinated (vax) and unvaccinated (no vax) SARS-CoV-2 infected patients. Data were analyzed using the Mann-Whitney U-test and the Wilcoxon signed-rank test for paired samples.


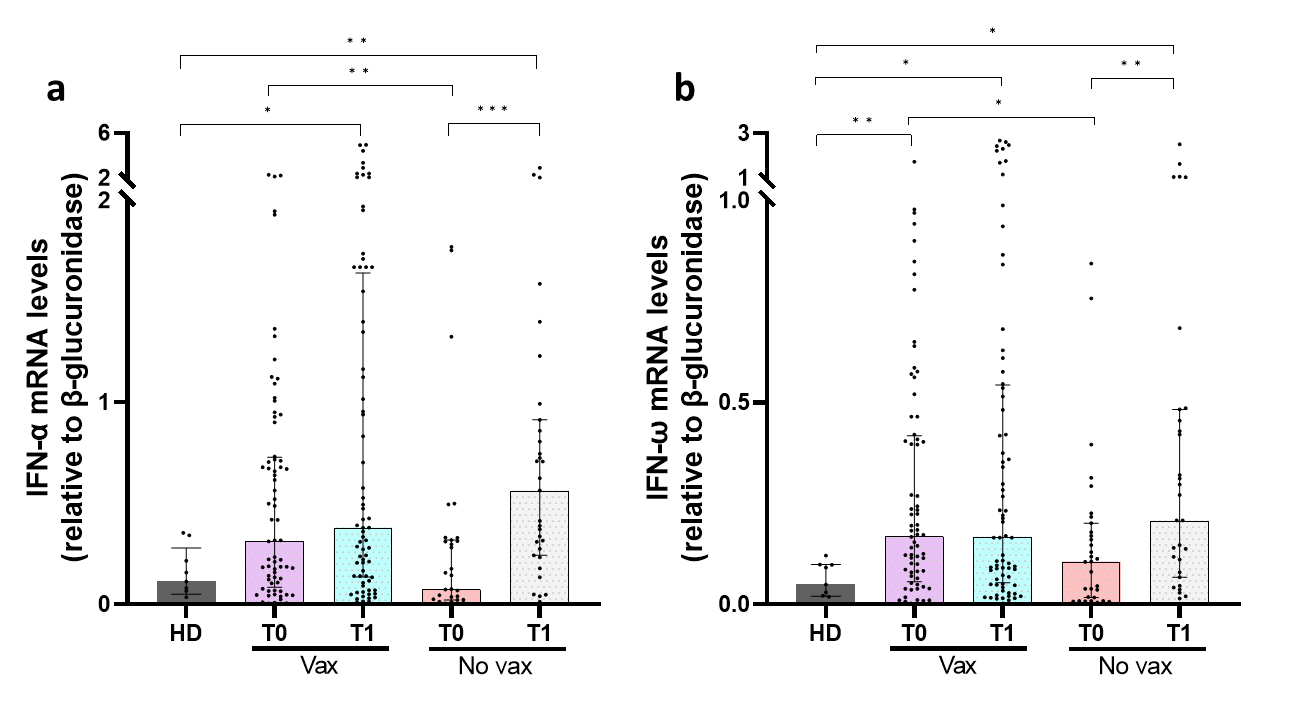


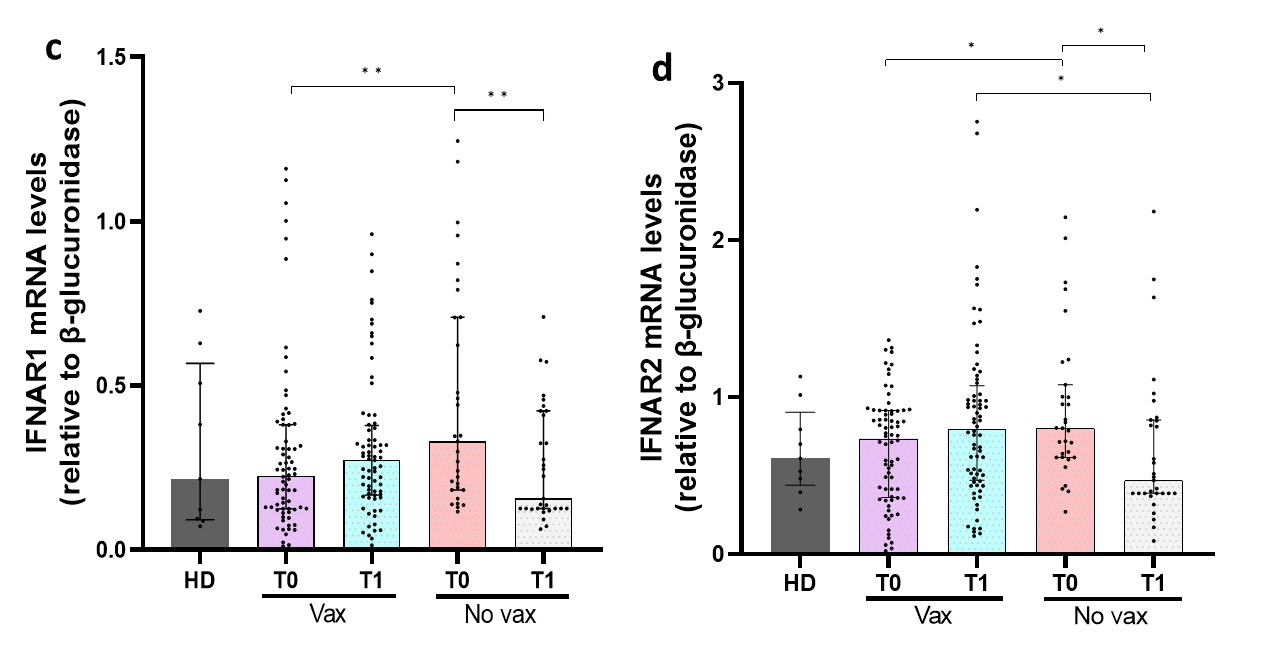


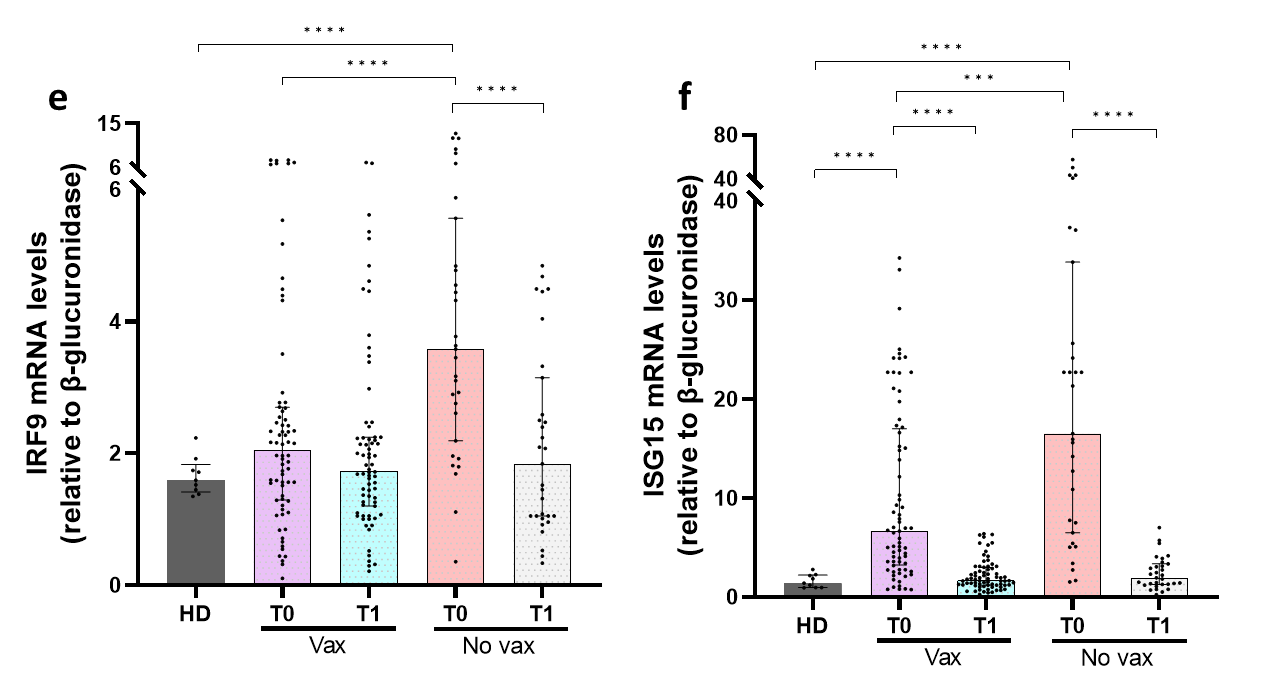


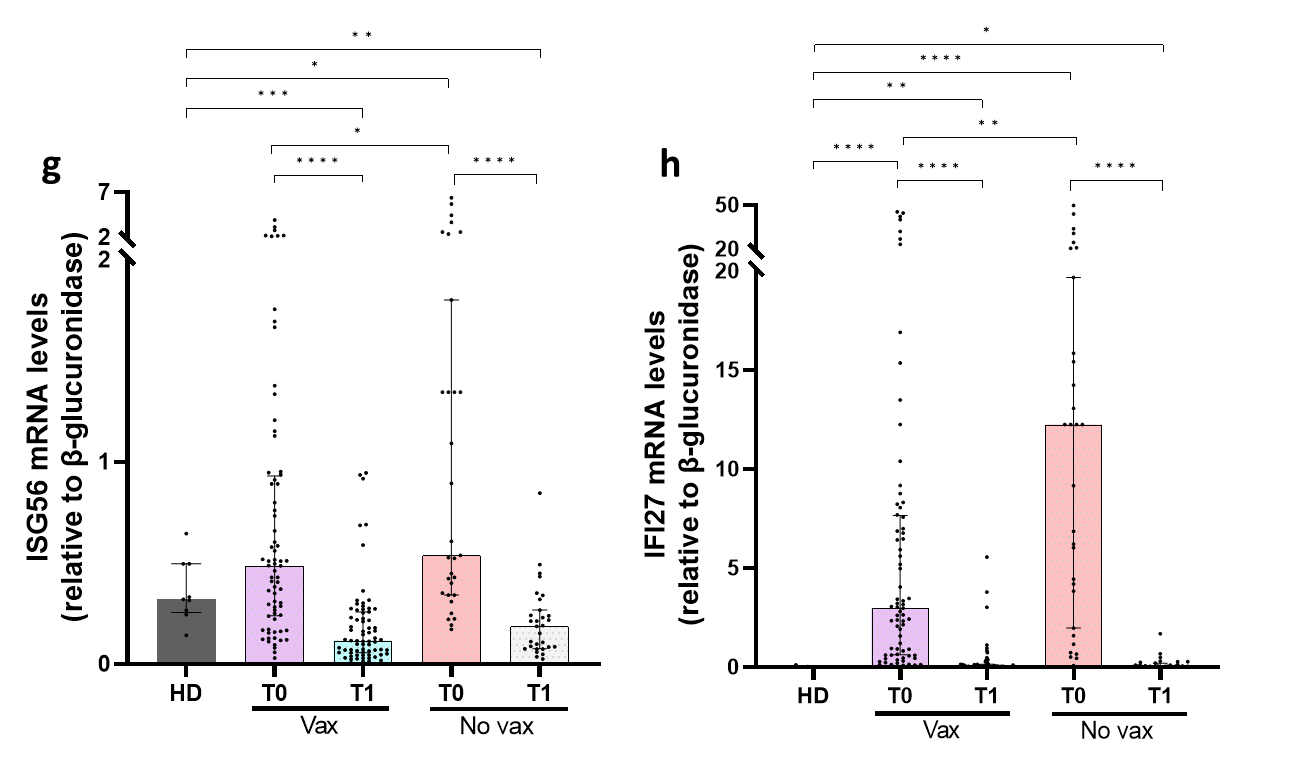


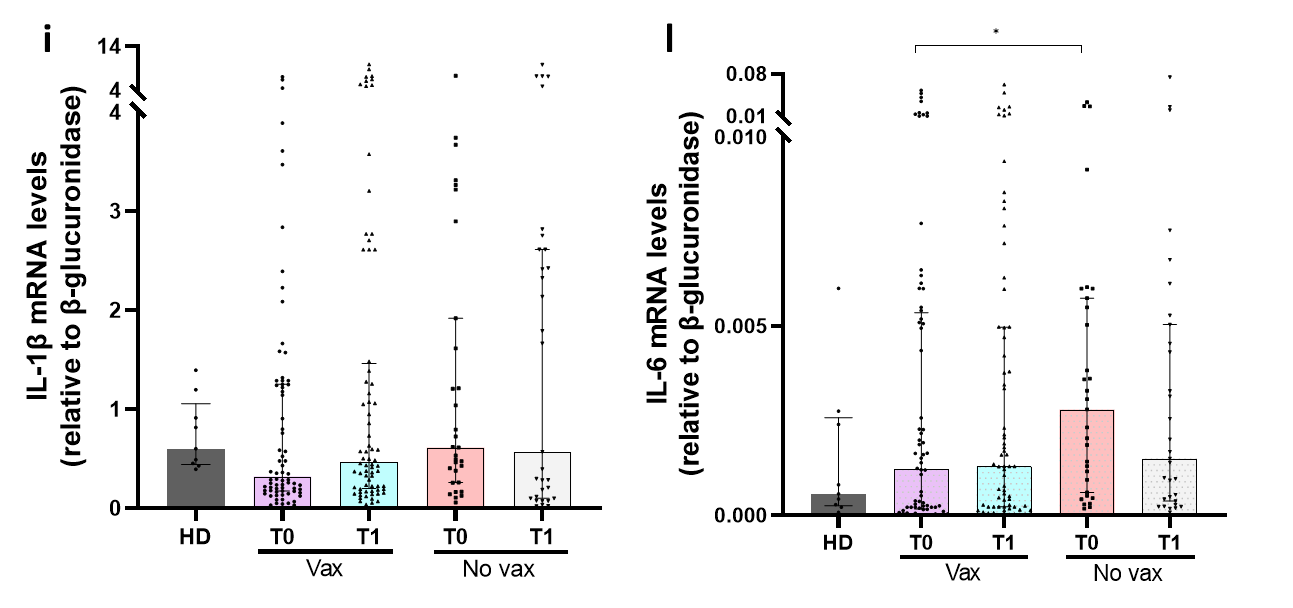


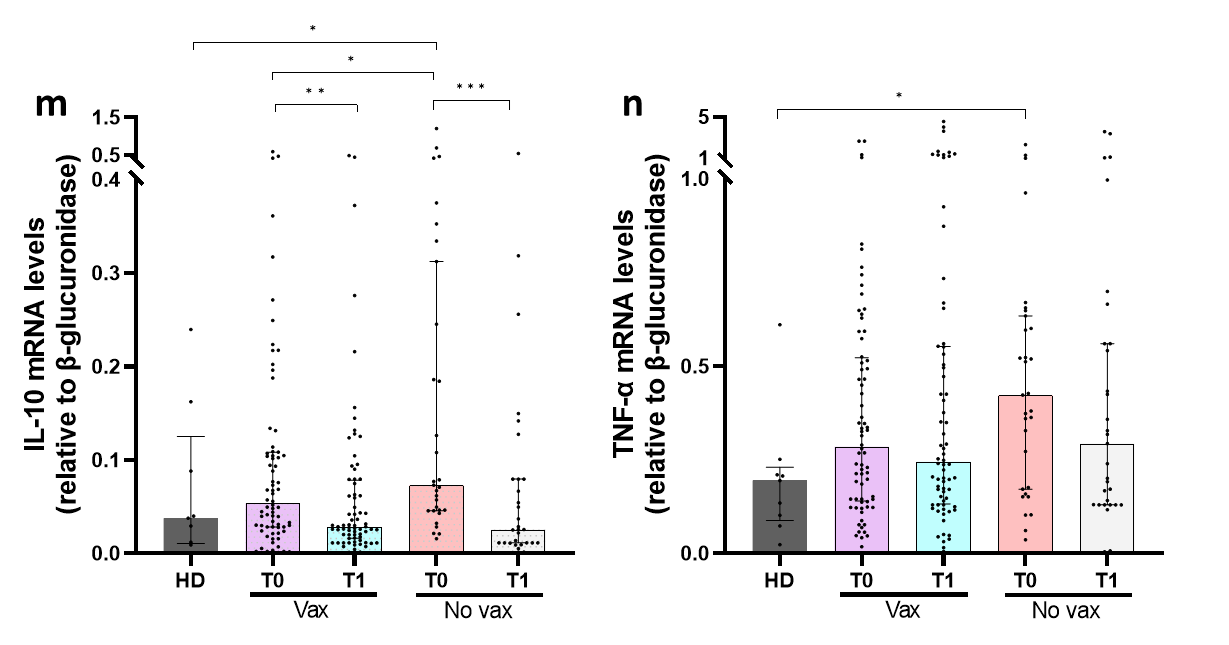


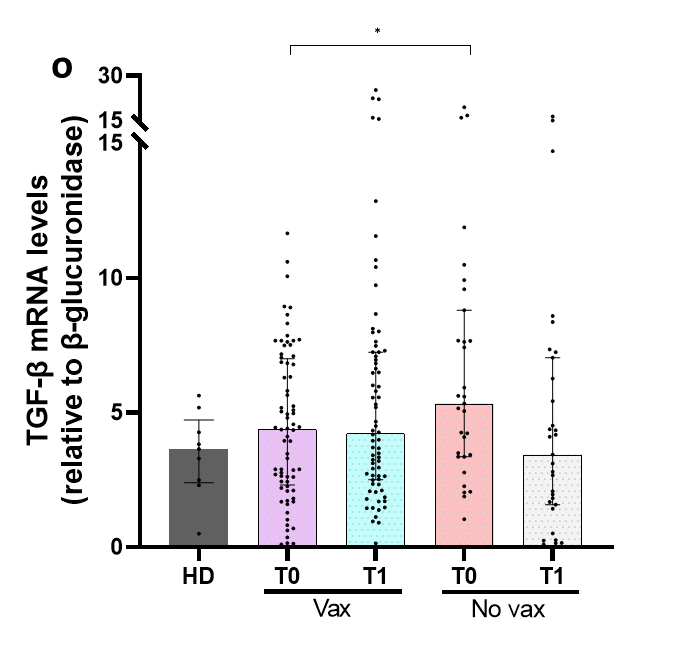


**Supplementary Figure 2** Comparison of IFN-α **(a),** IFN-ω **(b),** IFNAR1 **(c),** IFNAR2 **(d),** IRF9 **(e),** ISG15 **(f),** ISG56 **(g),** IFI27 **(h),** IL-1β **(i),** IL-6 **(l),** IL-10 **(m),** TNF-α **(n)** and TGF-β **(o)** mRNA expression levels between healty donors (HD) and vaccinated (vax) and unvaccinated (no vax) SARS-CoV-2 infected patients before (T0) and 12 days after mABs treatment (T1). Data were analyzed using the Mann-Whitney U-test and the Wilcoxon signed-rank test for paired samples.
